# SIRT1 and NRF2 Gene Transfer Mediate Distinct Neuroprotective Effects Upon Retinal Ganglion Cell Survival and Function in Experimental Optic Neuritis

**DOI:** 10.1167/iovs.17-22972

**Published:** 2018-03

**Authors:** Devin S. McDougald, Kimberly E. Dine, Alexandra U. Zezulin, Jean Bennett, Kenneth S. Shindler

**Affiliations:** Center for Advanced Retinal and Ocular Therapeutics, F. M. Kirby Center for Molecular Ophthalmology, Perelman School of Medicine, University of Pennsylvania, Philadelphia, Pennsylvania, United States

**Keywords:** AAV, neuroprotection, retinal ganglion cell, optic nerve, optic neuritis

## Abstract

**Purpose:**

Optic neuritis is a condition defined by autoimmune-mediated demyelination of the optic nerve and death of retinal ganglion cells. SIRT1 and NRF2 stimulate anti-inflammatory mechanisms and have previously demonstrated therapeutic value in preclinical models of neurodegenerative disease. Here we investigated the neuroprotective potential of SIRT1 or NRF2 gene transfer using adeno-associated virus (AAV) vectors in the experimental autoimmune encephalomyelitis (EAE) model of multiple sclerosis.

**Methods:**

C57Bl/6J mice were administered intravitreal doses of AAV2 vectors and immunized to induce EAE symptoms. Visual function was examined by recording the optokinetic response (OKR) just prior to EAE induction and once every 7 days postinduction for 7 weeks. Retina and optic nerves were harvested to investigate retinal ganglion cell survival (immunolabeling with Brn3a antibodies); inflammation (hematoxylin and eosin staining); and demyelination (luxol fast blue staining).

**Results:**

Animals modeling EAE demonstrate reduced visual acuity compared to sham-induced controls. Intravitreal delivery of AAV2-NRF2 did not preserve visual function. However, AAV2-SIRT1 mediated significant preservation of the OKR compared to AAV2-eGFP controls. Treatment with AAV2-NRF2 promoted RGC survival while AAV2-SIRT1 mediated an upward trend in protection compared to vehicle and AAV2-eGFP controls. Neither NRF2 nor SIRT1 gene augmentation was able to suppress optic nerve inflammation or demyelination.

**Conclusions:**

AAV-mediated overexpression of NRF2 or SIRT1 within RGCs mediates distinct neuroprotective effects upon visual function and RGC survival. This study expands our understanding of SIRT1 and NRF2-mediated neuroprotection in the context of MS pathogenesis and optic neuropathies.

Multiple sclerosis (MS) is a chronic neuroinflammatory disease characterized by CNS infiltration of autoimmune effector cells that mediate cytotoxic responses against conserved elements of neuronal tissue, including myelin and oligodendrocytes. Synaptic disjunction and subsequent neuronal apoptosis generates a clinical phenotype of progressive neurologic dysfunction.^1^ Patients often present with episodes of visual impairment due to optic neuritis, which involves acute inflammation and demyelination of the optic nerve.^2–4^ While vision typically improves several weeks after onset of optic neuritis, up to 60% of patients develop some level of permanent visual dysfunction due to optic nerve atrophy.^5^ Permanent vision loss correlates with degeneration of retinal ganglion cells (RGCs).^2–4^ Current treatments for MS include immunomodulatory agents to mitigate acute inflammatory episodes. Unfortunately, these interventions have limited effects on neurodegenerative processes that drive progressive neurologic dysfunction.^5^ Therefore, alternative therapeutic strategies that target conserved features of neuropathology while modulating the inflammatory response are greatly needed.

Current understanding of MS pathogenesis has benefited from studies of experimental autoimmune encephalomyelitis (EAE).^6^ Manifestations of optic neuritis in EAE include optic nerve thinning, RGC loss, and reduced visual function, similar to the human disease, thus providing an in vivo system for characterization of neurodegenerative processes and a platform for interrogating neuroprotective strategies.^7–9^ Prior studies suggest oxidative stress and mitochondrial injury are central mediators of MS pathology.^1^ Accumulation of reactive oxygen and nitrogen species (ROS/RNS) within MS lesions leads to damage of cellular components including proteins, lipids, and DNA. Fortunately, eukaryotic cells are equipped with a collection of defense systems to combat oxidative injury and maintain redox homeostasis.^10,11^ We hypothesized that amplifying such mechanisms within RGCs using a conventional gene transfer approach may promote neuroprotection in experimental optic neuritis.

Nuclear factor (erythroid-derived 2)-like 2 (NRF2) is a basic leucine zipper transcription factor that activates a network of genes associated with antioxidant defense and cellular detoxification.^11^ Transgenic ablation of *NRF2* during EAE development generates a phenotype of accelerated demyelination, immune cell infiltration, and proinflammatory cytokine signaling compared to wild-type animals also subjected to EAE.^12^ In addition, *Nrf2* knockout mice demonstrate enhanced decline in visual function, loss of RGCs, and exacerbated optic nerve atrophy.^13^ Accumulating evidence supports therapeutic modulation of NRF2 activity via small molecule activation or transgenic overexpression in neurodegenerative diseases driven by oxidation.^14–17^ A gene augmentation strategy demonstrated a transient increase in survival of RGCs targeted with NRF2 expression vectors following optic nerve crush.^17^ Based on the collective data, we reasoned that stimulation of NRF2 activity may provide an effective means to protect RGCs in optic neuritis.

Sirtuin 1 (SIRT1) is an evolutionarily conserved NAD^+^-dependent deacetylase that regulates various components of cellular metabolism with respect to aging, DNA repair, mitochondrial biogenesis, and apoptosis.^18^ Accumulating evidence suggests modulation of SIRT1 activity via pharmacologic induction or transgenic overexpression may offer therapeutic value in several forms of neurodegenerative disease.^19–27^ In experimental optic neuritis, small molecule activators of SIRT1, including resveratrol and related polyphenolic compounds, are effective in preserving visual acuity and RGC survival during EAE and viral-induced demyelinating disease.^19,21^ In addition, Nimmagadda et al.^24^ demonstrated suppression of inflammation and demyelination following EAE sensitization using a transgenic mouse containing neural-restricted overexpression of SIRT1. However, the study design was limited to MS lesions localized to the spinal cord and did not examine the contribution of SIRT1 overexpression in ameliorating ocular disease manifestations.^24^ Potential neuroprotective effects of SIRT1 overexpression specifically in RGCs need to be examined.

In the present study, we interrogated the effects of SIRT1 or NRF2 overexpression in experimental optic neuritis via adeno-associated virus (AAV) gene transfer to RGCs. We developed and characterized AAV serotype 2 (AAV2) vectors that drive constitutive expression of human NRF2 and SIRT1 in vitro and in the mouse retina. We examined the neuroprotective contribution of SIRT1 and NRF2 gene augmentation in suppressing RGC death, optic nerve inflammation and demyelination, and vision loss in EAE mice.

## Methods

### Animals

C57Bl/6J mice were obtained from the Jackson Laboratory and raised in a 12-hour light/dark cycle. Animals were housed at the University of Pennsylvania in compliance with ARVO Statement for the Use of Animals in Ophthalmic and Vision Research as well as with institutional and federal regulations.

### AAV Vector Design and Production

Human *SIRT1* (transcript variant 1) and human *NRF2* (transcript variant 1) cDNA clones were obtained from Origene. Sequences were amplified with Q5 DNA polymerase (NEB) and cloned into an AAV expression plasmid using a commercial cloning kit (In-Fusion HD; Clontech Laboratories, Mountain View, CA, USA). Transgene expression was driven by a CAG promoter derived from pDRIVE-CAG (InvivoGen, San Diego, CA, USA). Both cDNA sequences contained a C-terminal 3xFLAG epitope tag that terminates into a bovine growth hormone (bGH) polyadenylation sequence. AAV expression cassettes were flanked by the AAV2 inverted terminal repeats. A proviral plasmid driving expression of enhanced green fluorescent protein (*eGFP*) was obtained from Jean Bennett, MD, PhD (University of Pennsylvania) and contains identical *cis* regulatory elements. AAV2-NRF2, AAV2-SIRT1, and AAV2-eGFP vectors were generated using previously described methods and purified with CsCl gradient by the CAROT research vector core at the University of Pennsylvania.^2^

### Cell Culture

ARPE-19 cells were supplied by ATCC (Manassas, VA, USA) and grown at 37°C with 5% CO_2_. Cells were maintained in Dulbecco's modified Eagle's medium: nutrient mixture F-12 (DMEM/F12; Gibco Laboratories, Gaithersburg, MD, USA) and supplemented with 10% fetal bovine serum (FBS) and 1% penicillin-streptomycin. 84-31 cells were provided by James Wilson, MD, PhD (University of Pennsylvania) and were cultured in medium (DMEM-GlutaMax; Gibco Laboratories) and supplemented with 10% FBS and 1% penicillin-streptomycin. We seeded 84-31 cells at a density of 350,000 cells and transduced with AAV2 vectors at a multiplicity of infection (MOI) of 100,000. Cells were harvested for expression analysis at 48 hours posttransduction. For AAV transduction in ARPE-19 cells, 150,000 cells were plated and transduced with AAV2 vectors at an MOI of 100,000. Cells were harvested for expression analysis at 72 hours posttransduction. Cells were rinsed with PBS and fixed in 4% paraformaldehyde for 15 minutes at room temperature. Afterwards, cells were blocked in 0.1% Triton X-100 and 1% bovine serum albumin (BSA) for 30 minutes at room temperature. Cells were incubated with primary antibody solution containing 1% BSA and rabbit anti-FLAG antibody (CST #14793; 1:200) for 1 hour at room temperature. Cells were washed with PBS and incubated in secondary antibody solution containing 1% BSA and goat anti-rabbit AlexaFluor-594 antibodies (1:500) for 1 hour at room temperature. Cells were removed from secondary incubation, washed in PBS, and mounted with (Fluoromount-G; Southern Biotech; Birmingham, AL, USA) containing DAPI.

### Quantitative Real-Time PCR (RT-qPCR)

RNA was isolated from 84-31 cells (provided by James Wilson) using the RNA kit (Macherey-Nagel Nucleospin RNA kit; Thermo Fisher Scientific, Waltham, MA, USA). First-strand cDNA synthesis was performed using 500 ng of total RNA with the first-strand synthesis system (SuperScript III; Thermo Fisher Scientific) according to manufacturer's protocol. Real-time PCR was performed with a commercial system (7500 Fast; Applied Biosystems, Foster City, CA, USA) using a PCR master mix (Power SYBR green; Invitrogen). The following primer sequences were used: 5′ CCACTCCTCCACCTTTGAC 3′ (human *GAPDH* Forward); 5′ ACCCTGTTGCTGTAGCCA 3′ (human *GAPDH* Reverse); GAGCTGGGGTGTCTGTTTCA (human *SIRT1* Forward); GGAAGTCTACAGCAAGGCGA (human *SIRT1* Reverse); GTCACATCGAGAGCCCAGTC (human *NRF2* Forward); and AGCTCCTCCCAAACTTGCTC (human *NRF2* Reverse). Relative gene expression was quantified with the ΔΔC_T_ method and normalized to *GAPDH*.

### Intravitreal Injections

We anesthetized 4-week-old mice by isoflurane inhalation. A 33½ gauge needle was used to create a small incision at the limbus. Afterward, a 10-μL Hamilton syringe (701 RN; Hamilton Company, Reno, NV, USA) attached to a 33-gauge blunt-end needle was inserted into the vitreous cavity with the needle tip placed directly above the optic nerve head. We dispensed 2 μL of AAV preparation containing approximately 1 × 10^10^ vector genomes were dispensed into each eye bilaterally. Vehicle treated eyes were injected with an equivalent volume of vector dilution buffer (0.001% Pluronic F68 in PBS). The two eyes of each mouse received different injections (vehicle, AAV2-NRF2, AAV2-SIRT1, or AAV2-eGFP) allowing each eye to serve as an independent experimental end point.

### Induction and Score of EAE

We anesthetized 8-week-old C57Bl/6 mice by isoflurane inhalation and injected at two sites subcutaneously with 200 μg of myelin oligodendrocyte glycoprotein peptide (MOG_35–55_; GenScript, Piscataway, NJ, USA) emulsified in antigen solution (Complete Freund's Adjuvant [CFA]; Difco Laboratories, Inc., Detroit, MI, USA) with 2.5 mg/mL mycobacterium tuberculosis (Difco Laboratories, Inc.). Control mice that were not induced for EAE were injected with an equal volume of PBS and CFA. All mice were given 200 ng pertussis toxin (List Biological, Campbell, CA, USA) in 0.1 mL of PBS by intraperitoneal injection at 0 hours and 48 hours postimmunization with MOG_35–55_. Clinical EAE was assessed using a previously described five-point scale^19^: no disease = 0; partial tail paralysis = 0.5; tail paralysis or waddling gait = 1.0; partial tail paralysis and waddling gait = 1.5; tail paralysis and waddling gait = 2.0; partial limb paralysis = 2.5; paralysis of one limb = 3.0; paralysis of one limb and partial paralysis of another = 3.5; paralysis of two limbs = 4.0; moribund state = 4.5; death = 5.0.

### Optokinetic Response Recordings (OKRs)

Visual function was assessed by measuring the OKR using commercial software and apparatus (OptoMotry; CerebralMechanics, Inc., Medicine Hat, AB, Canada) as previously described.^28^ OKR was determined as the highest spatial frequency where mice track a 100% contract grating that is projected at different spatial frequencies. Measurements were performed by an investigator blinded to the experimental treatments.

### Retinal Histology and RGC Quantification

Eyes were harvested and placed in 4% paraformaldehyde (PFA) overnight at 4°C. Eyes were washed in PBS followed by dissection of retinal cups. Tissues were permeabilized and blocked in 2% Triton X-100, 10% normal donkey serum, and PBS and then incubated with goat anti-Brn3a antibody (Santa Cruz Biotechnology, Dallas, TX, USA) diluted 1:100 at 4°C. Retinal cups were washed and then incubated in secondary antibody solution containing 2% Triton X-100, 10% normal donkey serum, and donkey anti-goat AlexaFluor 594 antibody (1:500 dilution). After washing, samples were prepared as flatmounts and mounted onto glass slides with an aqueous mounting medium (SouthernBiotech) containing 4′,6-diamidino-2-phenylindole (DAPI). RGCs were quantified as previously described.^7,19,25,26^ Briefly, retinal micrographs were recorded at ×40 magnification in 12 standard fields (1/6, 3/6, and 5/6 of the retinal radius from the center of the retina in each quadrant). Total RGC counts from the 12 fields per retinal sample covering a total area of 0.45 mm^2^/retina were recorded by an investigator masked to the experimental conditions using ImageJ software (http://imagej.nih.gov/ij/; provided in the public domain by the National Institutes of Health, Bethesda, MD, USA). Retinal cross-sections were incubated in blocking buffer containing PBS, 2% Triton X-100, and 10% normal donkey serum for 1 hour at room temperature. Next, sections were incubated in primary antibody solution containing the previously described components and a rabbit anti-FLAG antibody (CST #14793) at 1:100 dilutions overnight in a humidified chamber at room temperature. Sections were washed in PBS three times and incubated in secondary antibody solution containing donkey anti-rabbit AlexaFluor 488 antibody diluted at 1:200 for 2 hours at room temperature. Slides were then washed in PBS three times and mounted with aqueous mounting medium (SouthernBiotech) containing DAPI.

### Optic Nerve Histology and Scoring

Histologic staining and scoring was performed as in prior studies.^7–9,19–25^ Optic nerves were harvested, fixed in 4% PFA, and embedded in paraffin. Nerves were subsequently cut into 5-μm longitudinal sections. To examine immune cell infiltration, sections were stained with hematoxylin and eosin (H&E). Inflammation was scored by an investigator blinded to the experimental treatments, and nerves were graded on a 0 to 4 point scale: no infiltration = 0; mild cellular infiltration = 1; moderate infiltration = 2; severe infiltration = 3; massive infiltration = 4. Sections were stained with luxol fast blue (LFB) to assess myelination. These sections were graded on a 0 to 3 point scale: 0 = no demyelination; 1 = scattered foci of demyelination; 2 = prominent foci of demyelination; and 3 = large (confluent) areas of demyelination.

### Statistics

All data are represented as means ± SEM. Differences between treatment groups with respect to OKR responses, RGC quantification, and optic nerve histopathology were compared using a 1-way ANOVA followed by Tukey's honest significant difference test using statistical software (GraphPad Prism 7.0; GraphPad Software, Inc., La Jolla, CA, USA). Differences were considered statistically significant at *P* < 0.05.

## Results

### Design and Characterization of AAV2 Vectors

**Figure 1:**
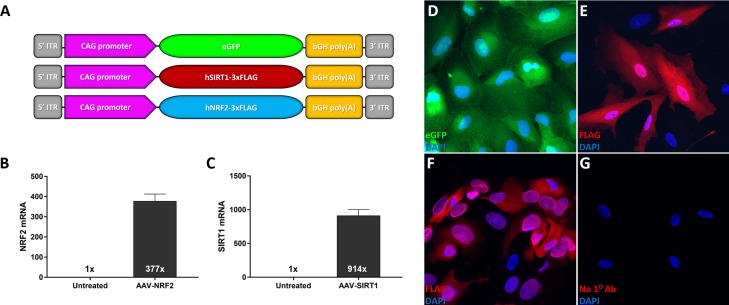
Design and in vitro characterization of AAV2 vectors. (A) Outline of AAV expression cassettes used in the study. RT-qPCR analysis of relative quantities of human NRF2 or human SIRT1 mRNA in 84-31 cells treated with (B) AAV2-NRF2 and (C) AAV2-SIRT1 compared to nontransduced cells. Micrographs displaying vector protein expression following ARPE-19 cell transduction with (D) AAV2-eGFP, (E) AAV2-NRF2, and (F) AAV2-SIRT1. (G) Control micrograph of ARPE-19 cells transduced with AAV2-NRF2 without anti-FLAG primary antibody incubation.

**Figure 2:**
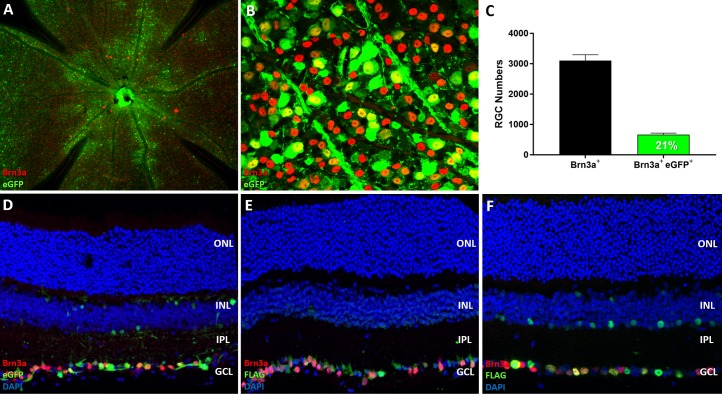
AAV2 transduction profile and RGC transduction efficiency following intravitreal delivery. (A) Representative micrograph of retinal flatmount following intravitreal injection of AAV2-eGFP. RGCs are labeled with Brn3a (red). (B) Representative visual field of a retinal flatmount used for calculating RGC transduction efficiency with AAV2. (C) Quantification of RGC transduction (n = 5 retina). (D) Representative cross-section of mouse retina following intravitreal injection of AAV2-eGFP, (E) AAV2-NRF2, and (F) AAV2-SIRT1. RGCs are labeled with Brn3a (red). Cells positively transduced with NRF2 or SIRT1 vectors are labeled with FLAG (green). Data represented as mean ± SEM.

Vectors based upon recombinant adeno-associated virus (AAV) have emerged as the current standard for achieving safe and stable gene transfer directed to nondividing cells such as neurons. AAV serotype 2 (AAV2) demonstrates a robust safety profile following subretinal delivery in clinical trials for Leber congenital amaurosis type 2.^29–32^ We generated AAV2 vectors expressing eGFP, human *NRF2*, or human *SIRT1* driven by a ubiquitous promoter (Fig. 1A). Vector expression was examined in vitro with RT-qPCR and immunofluorescence (Figs. 1B–H). RT-qPCR revealed robust levels of transgene expression in 84-31 cells treated with the designated vector compared to untreated controls (Figs. 1B, 1C). Immunofluorescent labeling of ARPE-19 cells transduced with AAV2-SIRT1 demonstrates strong nuclear localization of the transgene product (Fig. 1G), while cells transduced with AAV2-NRF2 display robust cytoplasmic and nuclear distribution of the tagged protein (Fig. 1F). Next, we examined the retinal transduction profile of AAV2 following intravitreal delivery with a vector expressing enhanced green fluorescent protein in a cohort of wild-type mice. Similar to previously described reports,^33–38^ AAV2-eGFP displayed transduction of the ganglion cell layer and optic nerve head (Figs. 2A, 2B). This vector achieved approximately 21% RGC transduction by quantifying the number of eGFP positive RGCs labeled with Brn3a antibody (Fig. 2C). AAV2 vectors driving expression of *NRF2* or *SIRT1* were injected into the right and left eyes, respectively, of wild-type mice display similar transduction profiles in vivo (Figs. 2D–F).

### *SIRT1* Gene Transfer Preserves RGC Function During EAE

**Figure 3:**
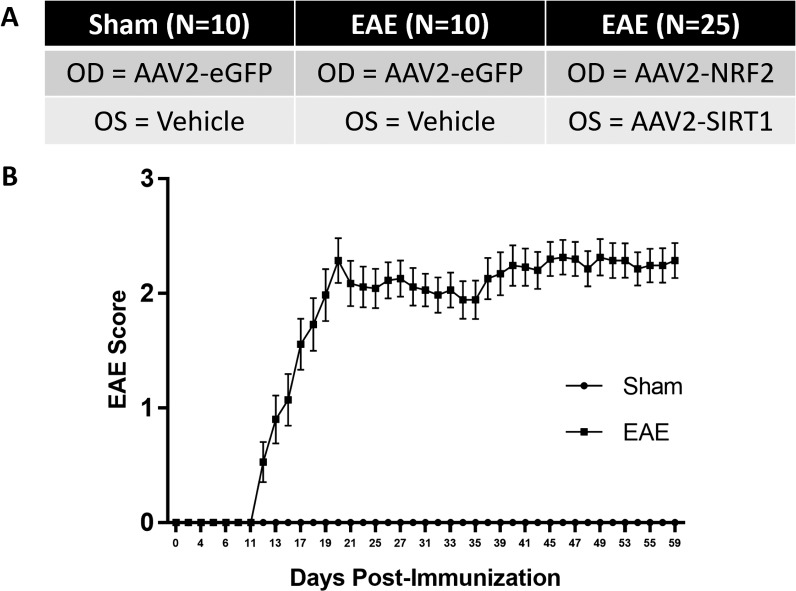
Experimental groups and clinical scoring of EAE. (A) Outline of the experimental groups used in the study. (B) Clinical scores of sham (n = 10) and EAE-induced (n = 35) animals. Data represented as mean ± SEM.

**Figure 4:**
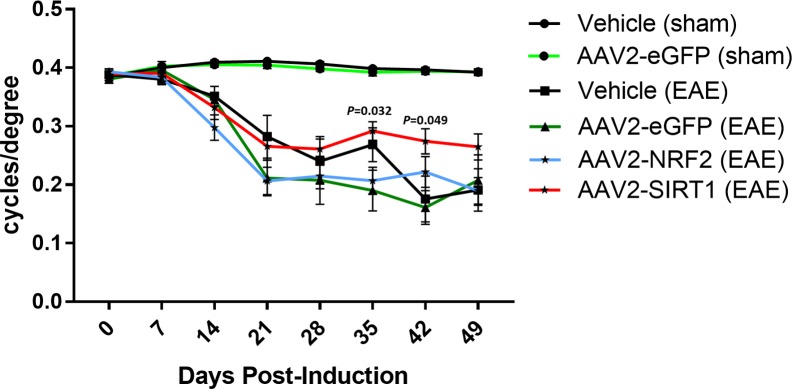
Effect of gene transfer on visual acuity during EAE. OKR recordings demonstrate significantly decreased visual acuity in eyes of EAE mice treated with vehicle (n = 1 0) or AAV2-eGFP (n = 10). Treatment with AAV2-NRF2 (n = 25) did not improve visual function. Mice treated with AAV2-SIRT1 (n = 25) show trending improvement in OKR at days 28 to 49 compared to EAE eyes injected with vehicle and significant improvement at days 35 (P = 0.032) and 42 (P = 0.049) compared to AAV2-eGFP injected eyes also subjected to EAE. Data represented as mean ± SEM. *P < 0.05, **P < 0.01 by 1-way ANOVA with Tukey's HSD post-test.

C57Bl6/J mice received intravitreal injections of AAV2 vectors or vehicle at postnatal week 4 followed by EAE/sham induction at postnatal week 8 (Fig. 3A). Following MOG_35–55_ immunization, animals displayed phenotypic features of EAE beginning near day 12 post-immunization (Fig. 3B) similar to prior studies.^7,19,25^ We measured visual function in response to gene transfer by recording the OKR prior to EAE/sham immunization and once every 7 days postimmunization. Earlier reports demonstrate a marked reduction in the OKR throughout the course of EAE.^7^ Sham-induced animals treated with intravitreal injections of vehicle or AAV2-eGFP exhibit robust OKR scores throughout the experimental timeline (Fig. 4), suggesting minimal adverse effects associated with intravitreal delivery, vector recruitment, and transgene overexpression. Similarly, animals injected with AAV2-NRF2 or AAV2-SIRT1 displayed strong responses prior to induction. Following EAE sensitization, MOG-induced animals exhibit a decline in OKR scores beginning by day 21 postinduction. However, eyes treated with AAV2-SIRT1 demonstrate an upward trend in functional responses throughout the experimental timeline. In addition, the AAV2-SIRT1 treatment group achieves statistically significant preservation at days 35 (AAV2-SIRT1 = 0.292 ± 0.016; AAV2-eGFP = 0.19 ± 0.035; *P* = 0.032) and 42 (AAV2-SIRT1 = 0.274 ± 0.022; AAV2-eGFP = 0.161 ± 0.029; *P* = 0.049) when compared to the EAE-induced control group treated with AAV2-eGFP. NRF2 augmentation did not provide statistically meaningful preservation of visual acuity throughout the experimental timeline.

### *NRF2* Gene Transfer Improves RGC Survival During EAE

**Figure 5:**
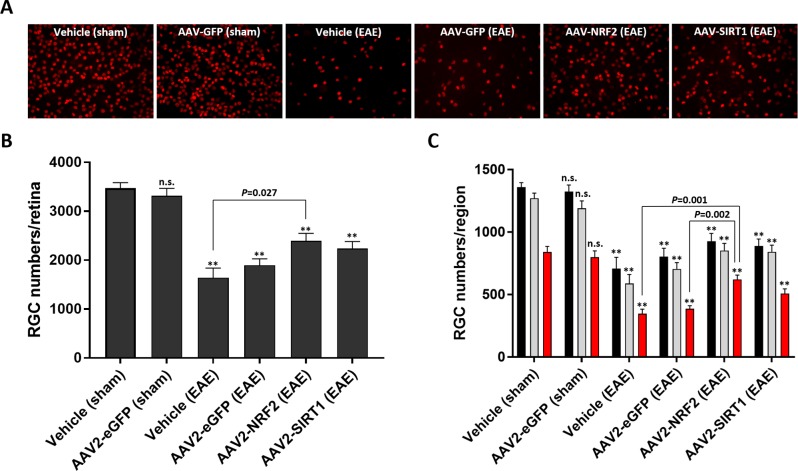
Effect of gene transfer on RGC survival during EAE. (A) Representative micrographs of RGC survival from each treatment group. RGCs are immunolabeled with Brn3a (red). (B) Total RGC numbers quantified per retina. (C) RGC numbers quantified per retinal region. Black bars: central. Gray bars: midperipheral. Red bars: peripheral. Data represented as mean ± SEM. *P < 0.05, **P < 0.01 by 1-way ANOVA and Tukey's HSD post-test.

Permanent visual decline in optic neuritis coincides with the loss of RGCs.^7^ Retinas from each treatment group were isolated and stained with antibodies directed against Brn3a, a marker of RGCs, to determine whether *SIRT1* or *NRF2* gene augmentation conferred a protective advantage upon RGCs during EAE (Fig. 5). Intravitreal injection of AAV2 was well tolerated as indicated by comparative total RGC counts in sham-induced animals treated with vehicle. In mice sensitized to EAE, RGC numbers were significantly reduced in all treatment groups compared to sham-induced controls injected with vehicle or AAV2-eGFP (*P* < 0.01). Treatment with AAV2-SIRT1 showed an upward trend in total RGC survival compared to control eyes, although this effect was not statistically significant. *NRF2* gene transfer did provide a statistically significant increase in RGC survival compared to eyes treated with vehicle (*P* = 0.027; Fig. 5B). We also examined the effect of *NRF2* or *SIRT1* gene transfer upon regional RGC density in the mouse retina (central, midperipheral, and peripheral; Fig. 5C). *NRF2* augmentation promoted survival of RGCs located within peripheral regions of the retina compared to both vehicle (*P* = 0.001) and AAV2-eGFP (*P* = 0.002) treatment groups sensitized to EAE. We observed a nonsignificant but trending increase in regional RGC density in retinas treated with AAV2-SIRT1.

### Gene Transfer With *NRF2* or *SIRT1* Fails to Attenuate Optic Nerve Inflammation and Demyelination

**Figure 6:**
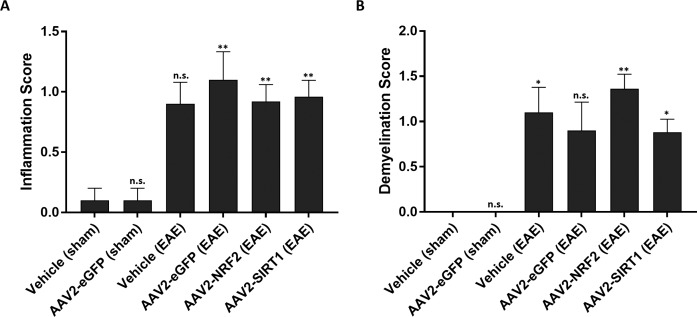
Effect of gene transfer on optic nerve inflammation and demyelination. (A) Inflammation scores quantified from optic nerve sections following H&E staining. (B) Demyelination scores from optic nerve sections following LFB staining. Numbers within columns indicate the number of nerves evaluated per group. Data represented as mean ± SEM. *P < 0.05, **P < 0.01 by 1-way ANOVA and Tukey's HSD post-test.

We investigated immune infiltration of the optic nerve in response to *SIRT1* or *NRF2* gene therapy. Optic nerve sections were subjected to H&E staining for evidence of immune cell infiltration. Optic nerves from sham-induced animals that received intravitreal injection of vehicle or AAV2-eGFP displayed minimal evidence of immune recruitment. However, all EAE-sensitized animal cohorts demonstrated enhanced infiltration. Optic nerves derived from animals dosed with *NRF2* or *SIRT1* vectors did not show a difference in immune recruitment compared to the vehicle and AAV2-eGFP treated animals undergoing EAE (Fig. 6A). We examined the effect of gene transfer on EAE-induced optic nerve demyelination by staining optic nerve sections with LFB. Sections from sham-induced animals injected with vehicle or AAV2-eGFP exhibited robust LFB labeling indicative of healthy nerves not subject to the immune-mediated demyelination of EAE. EAE animals treated with vehicle or AAV2-eGFP demonstrated decreased LFB staining compared to sham-induced animals. Gene transfer with *SIRT1* or *NRF2* did not prevent demyelination as these animals demonstrated similar myelination scores as EAE induced controls (Fig. 6B).

## Discussion

The present study explored the effects of SIRT1 or NRF2 gene transfer in experimental optic neuritis. Under cellular conditions of redox equilibrium, NRF2 is sequestered within the cytoplasm and subject to proteasomal-mediated degradation.^10,39,40^ During oxidative challenge, modifications to critical binding proteins free NRF2 to translocate into the nucleus, recruit transcriptional machinery to antioxidant response elements (AREs), and stimulate transcription of target genes associated with antioxidant defense and cellular detoxification.^11^ SIRT1 is recruited to the nucleus and other cellular compartments where it modulates the activity of various protein targets. SIRT1 is known to deacetylate and inhibit the transcription factor, p53, thereby downregulating apoptotic gene expression and thus improving cell viability.^41^ SIRT1 promotes mitochondrial function and antioxidant metabolism by activating PGC-1α, a master transcriptional regulator of these responses.^42^ While SIRT1 and NRF2 are typically believed to function via separate pathways, recent evidence suggests SIRT1 involvement in regulating the expression and activation of NRF2.^43^ In addition, treatment with pharmacologic agents such as resveratrol, a known activator of SIRT1, was shown to enhance NRF2 expression and activity of its downstream effectors.^44,45^ We hypothesized that gene augmentation of NRF2 or SIRT1 within RGCs could ameliorate pathologic features of experimental optic neuritis. Our data demonstrate distinct effects upon RGC survival and function following AAV2-mediated overexpression of NRF2 or SIRT1, suggesting these candidate factors promote neuroprotective mechanisms that may modify MS pathogenesis.

RGC-directed gene therapy with SIRT1 and NRF2 vectors revealed differential effects upon visual acuity during EAE. Visual acuity was not affected prior to EAE development with any of the vectors tested, suggesting vector delivery or transgene overexpression did not mediate unintended toxicity on retinal function. We observed a statistically significant decline in visual acuity beginning at day 21 postimmunization with all AAV2 and vehicle-treated animals subjected to EAE, whereas sham-induced cohorts presented robust responses throughout the experimental timeline. While Larabee et al.^13^ reported that *NRF2* knockout mice exhibit increased visual decline compared to wild-type cohorts during EAE, augmenting NRF2 activity with AAV2 gene transfer was unable to preserve visual acuity in the current study. Failure to reverse effects of knockout studies may be due to the limited number of RGCs (21%) infected with the AAV2 vectors in this study. However, interestingly, overexpression of SIRT1 mediated a trending increase in functional recovery beginning at day 28 postinduction compared to vehicle and AAV2-eGFP control groups subjected to EAE. This protective effect achieved statistical significance compared to the AAV2-eGFP control group at 35 and 42 days postinduction, which is remarkable given that only a subset of RGCs were transfected. This finding also correlates with prior investigations utilizing compounds that stimulate SIRT1 activity and demonstrate varying degrees of OKR preservation in the context of experimental optic neuritis as well as optic nerve crush.^19,26,27^

RGC numbers were significantly reduced in all animal groups sensitized to EAE. However, we observed increased RGC numbers with SIRT1 and NRF2 gene augmentation compared to the AAV2-eGFP and vehicle treatment groups. SIRT1 gene transfer did not mediate a statistically significant increase in RGC numbers but only a positive trend in survival compared to EAE-induced controls. NRF2 gene transfer provided the most robust protective response with respect to total and regional RGC survival. This outcome is particularly interesting as NRF2 augmentation did not correlate with an improvement in retinal function as shown by OKR recordings. However, disparities between OKR and RGC survival have been previously documented in this model.^27^ Another explanation for this finding could be that NRF2 overexpression is simply supporting survival of the RGC cells bodies but unable to sustain function. This interpretation is consistent with findings by Xiong et al.^17^ where NRF2 gene transfer–mediated transient yet significant preservation of RGCs following optic nerve crush without promoting axonal regeneration required for functional retention. Importantly, as indicated above, we only achieved approximately 21% RGC transduction with the AAV2 vector and previously described dose. Regarding the discrepancy between OKR preservation and RGC survival following AAV2-SIRT1 treatment, it is also possible that SIRT1 augmentation may influence the survival of ON direction-selective ganglion cells, which is the subset of cells that contribute to the OKR, but not mediate a statistically significant effect upon total RGC survival.^46^ Moreover, selection of a vector platform with enhanced capabilities for RGC transduction may provide a more potent means of cellular protection and functional preservation in this model. Recent developments utilizing rational design and in vivo selection have generated novel AAV capsids with improved potency and tropism for retinal cell types compared to naturally isolated serotypes such as AAV2.^47–51^ Further investigation into SIRT1 or NRF2-mediated neuroprotection in this model with an improved vector system is certainly warranted. The differential effects observed here also suggest a potential role for combined therapy with overexpression of both SIRT1 and NRF2. Due to limitations of the current transduction efficiency and the total volume that can be injected in the eye, coinjection of both vectors is not feasible, but future development of improved vector systems may allow investigation of a dual therapy.

While we did observe evidence of neuroprotection upon RGC function and viability, overexpression of neither NRF2 nor SIRT1 was able to suppress the inflammatory and demyelinating phenotype associated with optic neuritis. RGC-directed gene therapy did not influence immune recruitment to the optic nerve as shown by H&E histological analysis. This observation correlates with previous studies that examined small molecule-mediated neuroprotection during EAE. Specifically, pharmacologic activators of SIRT1, including resveratrol and related compounds, did not suppress inflammation in the spinal cord or optic nerve when administered at various doses in the same EAE model^19^ used in the current study. Interestingly, transgenic overexpression of human SIRT1 within neurons was able to reduce inflammation within spinal cord lesions.^24^ Similar to the effects on immunomodulation, AAV2-mediated expression of NRF2 or SIRT1 did not alleviate optic nerve demyelination. While these approaches did not attenuate demyelination, other studies that examined antioxidant or mitochondrial-directed gene therapy strategies during EAE have shown preserved myelin in the optic nerve.^34–38^ However, discrepancies in animal models, EAE immunization protocols, and other components of study design limit a direct comparison with these reports. In addition, our findings with respect to inflammation and myelination may once again reflect the limited transduction efficiency of the AAV2 vector.

Collectively, this study demonstrates at least partial neuroprotective effects of NRF2 and SIRT1 gene augmentation in the context of experimental optic neuritis, and suggests an important role of these signals in MS pathogenesis. Moreover, it underscores the therapeutic potential of targeting conserved cell survival pathways or mechanisms to impede progression of complex neurodegenerative disease.
